# Cytokine and genetic crosstalk in patients with coexistent familial Mediterranean fever and celiac disease: a cross-sectional study from Türkiye

**DOI:** 10.3389/fimmu.2026.1891366

**Published:** 2026-10-06

**Authors:** Fatih Öner Kaya, Haydar Kaan Karataş, Pınar Mert, Çağdaş Enginoğlu

**Affiliations:** Department of Internal Medicine, Maltepe University Faculty of Medicine, Training and Research Hospital, İstanbul, Türkiye

**Keywords:** autoinflammation, celiac disease, cytokine profile, familial Mediterranean fever, gene–gene interaction, HLA-DQ2/DQ8, MEFV mutation

## Abstract

**Background:**

Familial Mediterranean Fever (FMF) and Celiac Disease (CD) are two distinct inflammatory disorders that arise through different immunological pathways, yet they show a notable tendency to coexist, particularly in Mediterranean populations. The immunological consequences of this coexistence have not been adequately characterized.

**Objective:**

We set out to compare serum cytokine profiles and explore potential genetic interactions in patients with both FMF and CD versus those with FMF alone or CD alone. One hundred adult patients were enrolled and divided into three groups: 40 with coexistent FMF and CD, 30 with FMF only, and 30 with CD only. Serum concentrations of IL-1β, IL-6, TNF-α, and IL-10 were quantified by ELISA; CRP and SAA were measured using routine laboratory methods. MEFV mutations and HLA-DQ2/DQ8 genotypes were determined by PCR. Statistical evaluations included one-way ANOVA with Bonferroni correction, multivariable linear regression incorporating a formal interaction term, and principal component analysis to visualize cytokine clustering patterns.

**Results:**

Patients with both diseases exhibited significantly higher serum levels of IL-1β, IL-6, TNF-α, and CRP than those in either single-disease group (all p < 0.001), while IL-10 levels were notably lower (p = 0.02). Serum amyloid A levels were also significantly elevated in the coexistence group compared with both single-disease groups (p < 0.001) and correlated strongly with CRP (r = 0.69, p < 0.001). IL-6 emerged as the strongest independent predictor of CRP elevation (adjusted β = 0.47, 95% CI 0.31–0.63, p < 0.001). Principal component analysis identified a distinct pro-inflammatory cytokine cluster unique to the coexistence group. The simultaneous presence of HLA-DQ2 and the MEFV M694V variant was associated with a significant rise in IL-6 production (p < 0.001).

**Conclusion:**

When FMF and CD coexist, the resulting inflammatory state is substantially amplified and dysregulated, characterized by markedly elevated IL-6 and associated with a possible interaction between MEFV and HLA loci that requires confirmation in larger, independently sampled cohorts. These observations emphasize the importance of recognizing this overlap syndrome in clinical practice.

## 1 Introduction

Patients carrying diagnoses of both Familial Mediterranean Fever and Celiac Disease have been observed to present with more pronounced symptoms and greater laboratory evidence of systemic inflammation than those with either condition alone, suggesting that the coexistence of these disorders may produce an amplified inflammatory state beyond what each disease generates independently. FMF is a prototypic autoinflammatory disease triggered by mutations in the MEFV gene that impair the regulatory function of pyrin, resulting in uncontrolled inflammasome activation and excessive release of IL-1β (1–3). By contrast, Celiac Disease represents a gluten-driven autoimmune enteropathy that develops in genetically susceptible individuals who carry HLA-DQ2 or HLA-DQ8 alleles and is characterized by a predominant Th1-type mucosal immune response (4–6).

Although the initiating mechanisms differ—one rooted in innate immunity and the other in adaptive immunity—the two disorders converge on several shared downstream inflammatory mediators, most prominently IL-1β and IL-6 (7, 8). Over recent years, epidemiological observations from Mediterranean regions have indicated that FMF and CD occur together more frequently than would be predicted by chance (9–12). This overlap prompted us to consider whether MEFV mutations might lower the threshold for effective antigen presentation in HLA-DQ2/DQ8-positive individuals, thereby intensifying and sustaining the adaptive immune response (13).

Despite these suggestive clinical and epidemiological hints, systematic studies examining the combined cytokine signature and genetic interactions in patients with both conditions remain scarce. We therefore designed the present cross-sectional study to address this gap. Our primary goals were to compare circulating cytokine profiles across the three patient groups, to formally evaluate potential interactions between key MEFV and HLA variants, and to gain a broader view of the overall inflammatory landscape using principal component analysis.

## 2 Materials and methods

### 2.1 Study design and ethical approval

This cross-sectional study was performed in the Department of Internal Medicine at Maltepe University Faculty of Medicine, İstanbul, Türkiye, between January 2022 and December 2024. The institutional ethics committee granted approval (no. 2022/134), and the study was conducted in full accordance with the Declaration of Helsinki. Written informed consent was obtained from every participant prior to enrolment. The reporting of the study adhered to the STROBE guidelines for observational research.

### 2.2 Participants

A total of 100 adult patients were recruited and allocated to three groups: coexistent FMF and CD (n = 40), FMF only (n = 30), and CD only (n = 30). The diagnosis of FMF was established according to the Tel-Hashomer criteria (14), while CD required both positive anti-tissue transglutaminase serology and compatible histopathological findings on duodenal biopsy (15). To reduce potential confounding, we restricted inclusion to clinically stable individuals: FMF patients had been attack-free for at least eight weeks, and CD patients had maintained a strict gluten-free diet for a minimum of six months. Individuals with active infection, concomitant inflammatory bowel disease, other autoimmune disorders, malignancy, or current systemic immunosuppressive treatment were excluded. Demographic characteristics and ongoing treatment details—especially colchicine use (16)—were systematically recorded. Disease duration was collected where available but could not be uniformly verified across all participants and was therefore excluded from the primary analysis.

### 2.3 Sample size calculation

Power analysis was performed with G*Power software (version 3.1) (17). Assuming a medium effect size (Cohen’s f = 0.25), α = 0.05, and 80% statistical power for between-group comparisons, a minimum of 84 participants was required. We enrolled 100 patients to provide adequate power for multivariable regression modeling and exploratory subgroup analyses. The original sample-size calculation was based on the primary between-group comparison. Regression and PCA analyses were exploratory in nature. Although the overall participant-to-predictor ratio was considered acceptable, the interaction analysis was limited by the small and unevenly distributed dual-positive subgroup and should be interpreted as hypothesis-generating rather than confirmatory.

### 2.4 Laboratory and genetic analyses

Fasting venous blood samples were obtained, centrifuged within 30 minutes of collection, aliquoted, and stored at −80 °C until batch analysis; a single freeze-thaw cycle was applied. Serum IL-1β, IL-6, TNF-α, and IL-10 concentrations were measured in duplicate using commercially available ELISA kits (Bio-Rad Laboratories, Hercules, CA, USA) according to the manufacturer’s instructions. All cytokine assays were performed in a single batch to minimise inter-assay variability. CRP levels were quantified by automated immunoturbidimetry. Serum amyloid A (SAA) was quantified by nephelometry using a routine clinical laboratory platform. Laboratory personnel remained blinded to clinical group assignments throughout the analytical process.

Genomic DNA was extracted from peripheral blood leukocytes with a standard commercial kit. The five most clinically relevant MEFV variants (M694V, M680I, V726A, E148Q, R761H) were genotyped by sequence-specific PCR. Compound heterozygosity was defined as carriage of two different pathogenic MEFV variants (e.g., M694V/V726A, M694V/E148Q, or M694V/M680I) (18–20). HLA-DQ2/DQ8 alleles were determined by established PCR-based methods (15). All genotype distributions were verified against Hardy–Weinberg equilibrium expectations.

### 2.5 Statistical analysis

Statistical analyses were conducted using SPSS version 26.0 and Python’s Statsmodels library (21). Continuous variables are presented as mean ± standard deviation and categorical variables as frequencies and percentages. Normality was assessed with the Shapiro–Wilk test and homogeneity of variances with Levene’s test. Group comparisons were performed with one-way ANOVA followed by Bonferroni *post-hoc* correction when parametric assumptions were met; otherwise the Kruskal–Wallis test was applied. Effect sizes were expressed as eta squared (η²).

Multivariable linear regression models were constructed with CRP and IL-6 as dependent variables, adjusting for age, sex, and colchicine use. A specific interaction term (HLA-DQ2 × M694V) was introduced to evaluate potential interaction effects between genetic variants. Model diagnostics included variance inflation factors and adjusted R². Principal component analysis was performed on the cytokine dataset after confirming sampling adequacy with the Kaiser–Meyer–Olkin measure and Bartlett’s test of sphericity (22), retaining components with eigenvalues > 1. A two-sided p-value < 0.05 was considered statistically significant.

## 3 Results

### 3.1 Demographic and genetic characteristics

The three study groups were well balanced with regard to age and sex distribution (**Table 1**). HLA-DQ2 positivity was substantially higher in both the coexistence and CD-only groups compared with the FMF-only group. Among FMF-containing groups, M694V homozygosity was observed exclusively in the FMF + CD group (10/40, 25%), whereas no FMF-only patient was homozygous. Compound heterozygous genotypes were identified in 16 (40%) FMF + CD patients (M694V/V726A and M694V/E148Q) and 11 (37%) FMF-only patients (M694V/M680I). A simple heterozygous M694V genotype was present in 14 (35%) and 14 (47%) patients in the FMF + CD and FMF-only groups, respectively. Five FMF-only patients (17%) had no detectable MEFV mutation. Colchicine was used by all FMF-containing patients; mean daily dose was higher in the FMF + CD group (1.45 ± 0.39 mg/day) than in the FMF-only group (1.18 ± 0.25 mg/day). No patient in either FMF-containing group required anti-interleukin-1 therapy. No celiac patient required additional pharmacological treatment beyond a gluten-free diet (23, 24). Notably, 25% of patients with both diseases carried the combination of HLA-DQ2 and M694V—a pattern that was absent in the single-disease groups.

**Table 1:** Demographic and selected genetic features of the study groups.

Characteristic	FMF + CD (n=40)	FMF only (n=30)	CD only (n=30)	P-value
Age, years (mean ± SD)	35.2 ± 9.8	34.5 ± 10.5	34.1 ± 10.8	0.87
Female, n (%)	24 (60)	16 (53)	18 (60)	0.72
Colchicine use, n (%)	40 (100)	30 (100)	0 (0)	<0.001
Colchicine dose, mg/day (mean ± SD)	1.45 ± 0.39	1.18 ± 0.25	—	<0.001
HLA-DQ2 positive, n (%)	31 (78)	4 (13)	24 (80)	<0.001
HLA-DQ8 positive, n (%)	13 (32)	2 (7)	10 (33)	0.012
M694V homozygous, n (%)	10 (25)	0 (0)	0	
M694V heterozygous, n (%)	14 (35)	14 (47)	0	
Compound heterozygous, n (%)	16 (40)	11 (37)	0	
No detectable MEFV mutation, n (%)	0 (0)	5 (17)	—	
Dual HLA-DQ2 + M694V, n (%)	10 (25)	0	0	<0.001

### 3.2 Cytokine and inflammatory marker levels

Patients with coexistent FMF and CD displayed a distinctly heightened inflammatory profile. Serum concentrations of IL-1β, IL-6, TNF-α, and CRP were all significantly elevated compared with the single-disease groups (all p < 0.001). In contrast, IL-10 levels were significantly reduced in the coexistence group (p = 0.02) (25). IL-6 exhibited the largest between-group difference (η² = 0.32), underlining its central position in the amplified inflammatory response. Serum amyloid A (SAA) levels were also significantly elevated in the FMF + CD group (44.8 ± 14.1 mg/L) compared with FMF only (18.6 ± 6.2 mg/L) and CD only (14.5 ± 4.6 mg/L; all p < 0.001, η² = 0.67), and showed a strong positive correlation with CRP across all groups (r = 0.69, p < 0.001) (**Table 2**).

**Table 2:** Serum cytokine and inflammatory marker concentrations across the study groups.

Parameter	FMF + CD (n=40)	FMF only (n=30)	CD only (n=30)	P-value	η²
IL-1β (pg/mL)	12.4 ± 3.8	6.2 ± 2.1	5.8 ± 1.9	<0.001	0.25
IL-6 (pg/mL)	25.6 ± 7.2	11.3 ± 4.5	9.8 ± 3.9	<0.001	0.32
TNF-α (pg/mL)	18.7 ± 5.4	9.5 ± 3.2	8.9 ± 2.8	<0.001	0.28
IL-10 (pg/mL)	4.1 ± 1.6	6.8 ± 2.3	7.2 ± 2.1	0.02	0.11
CRP (mg/L)	8.9 ± 3.1	4.2 ± 1.8	3.7 ± 1.5	<0.001	0.29
SAA (mg/L)	44.8 ± 14.1	18.6 ± 6.2	14.5 ± 4.6	<0.001	0.67

Data are presented as mean ± standard deviation. All cytokine distributions passed the Shapiro–Wilk normality test (all p > 0.05), supporting the use of parametric tests. p-values were calculated using one-way ANOVA with Bonferroni *post-hoc* correction. Pairwise comparisons (Bonferroni-adjusted): for IL-1β, IL-6, TNF-α, CRP, and SAA, FMF + CD was significantly higher than both FMF only and CD only (all p < 0.01); no significant difference was observed between FMF only and CD only for these parameters. For IL-10, FMF + CD was significantly lower than both FMF only and CD only (both p < 0.001); FMF only and CD only did not differ significantly. For SAA, all three pairwise comparisons were significant (all p < 0.05). Effect sizes are expressed as eta squared (η²): small ≥ 0.01, medium ≥ 0.06, large ≥ 0.14. IL, interleukin; TNF, tumor necrosis factor; CRP, C-reactive protein; SAA, serum amyloid A; FMF, Familial Mediterranean Fever; CD, Celiac Disease.

### 3.3 Correlations and regression analysis

Bivariate correlation analysis demonstrated a strong positive relationship between IL-6 and CRP (r = 0.59, p < 0.001) and between SAA and CRP (r = 0.69, p < 0.001). In the fully adjusted multivariable model, IL-6 remained the most powerful independent predictor of CRP elevation (adjusted β = 0.47, 95% CI 0.31–0.63, p < 0.001), explaining a substantial proportion of the variance (adjusted R² = 0.61). Lower IL-10 concentrations and carriage of both HLA-DQ2 and M694V were also independently associated with higher CRP levels (26, 27). As detailed in the newly integrated **Table 3**, all variance inflation factors (VIF) remained strictly below 2.0, ruling out significant multicollinearity. The formal interaction term revealed a statistically significant association between the co-occurrence of HLA-DQ2 and M694V and elevated IL-6 production (β = 0.38, p < 0.001). Given that this genotype combination was observed exclusively in the FMF + CD group, this finding should be interpreted with caution, as it may partly reflect group membership rather than an independent gene–gene effect.

**Table 3:** Multivariable linear regression analysis for predictors of systemic inflammation (CRP levels).

Predictor variable	Unstandardized coefficient (B)	Standardized coefficient (β)	95% confidence interval (CI)	P-value	Variance inflation factor (VIF)
Intercept	2.14	—	1.05 – 3.23	0.002	—
IL-6 (pg/mL)	**0.21**	**0.47**	**0.31 – 0.63**	**< 0.001**	1.42
IL-10 (pg/mL)	-0.34	-0.19	-0.52 – -0.16	0.012	1.28
HLA-DQ2 × *MEFV* M694V	**1.85**	**0.38**	**0.19 – 0.57**	**< 0.001**	1.61
Age (years)	0.02	0.05	-0.04 – 0.08	0.540	1.12
Sex (Female)	0.11	0.03	-0.22 – 0.44	0.712	1.08
Colchicine Use	-0.45	-0.12	-0.98 – 0.08	0.094	1.35

• Model diagnostics: Overall Model Fit: Adjusted R² **=** 0.61; Model Significance: F = 24.85, p < 0.001.

• CRP, C-reactive protein; IL, interleukin; VIF, variance inflation factor.

• The formal interaction term (HLA-DQ2 × M694V) represents carriers harboring both variants simultaneously, exclusive to the FMF + CD group, and should be interpreted strictly as hypothesis-generating.

Bold values highlight the regression estimates of predictors with p < 0.001.

### 3.4 Principal component analysis

Principal component analysis provided clear visual separation of the coexistence group from the single-disease groups (22). The first principal component (explaining 62% of total variance) loaded heavily on IL-6, TNF-α, IL-1β, and CRP, reflecting a dominant pro-inflammatory axis. The second principal component accounted for 18% of variance and was largely driven by IL-10, suggesting a partially independent regulatory dimension. The Kaiser–Meyer–Olkin value of 0.73 and a significant Bartlett’s test of sphericity (p < 0.001) confirmed the suitability of the dataset for factor analysis.

**Figure 1** Principal component analysis score plot illustrating the distinct clustering of the FMF + CD group (red dots) separate from the FMF-only (green triangles) and CD-only (blue squares) cohorts. PC1 (x-axis, 62% variance) represents the pro-inflammatory axis; PC2 (y-axis, 18% variance) represents the regulatory axis.

**Figure 1:**
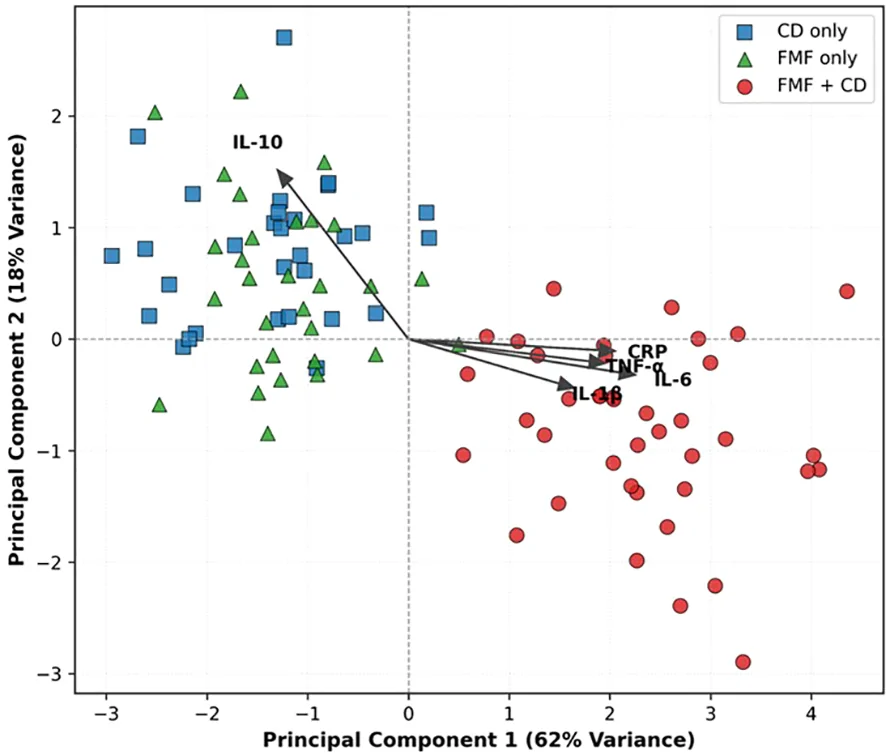
Principal component analysis (PCA) score plot illustrating the distinct clustering of the FMF + CD group (red dots) separate from the FMF-only (green triangles) and CD-only (blue squares) cohorts. The first principal component (PC1, x-axis) accounts for 62% of total variance and loads heavily on IL-6, TNF-α, IL-1β, and CRP, reflecting a dominant pro-inflammatory axis. The second principal component (PC2, y-axis) accounts for 18% of variance and reflects IL-10 loading, representing a partially independent regulatory dimension. Each data point represents one patient. FMF, Familial Mediterranean Fever; CD, Celiac Disease; PC, principal component.

## 4 Discussion

When we examined the data, one observation stood out sharply: the coexistence of FMF and Celiac Disease appears to be associated with an inflammatory response that exceeds what might be expected from either condition alone. Patients with both conditions had consistently higher levels of the key pro-inflammatory cytokines and CRP, along with a clear reduction in the regulatory cytokine IL-10 (8, 28).

We were particularly interested in the genetic interaction between MEFV and HLA loci. The M694V variant leads to a gain-of-function change in pyrin, causing exaggerated inflammasome assembly, increased caspase-1 activity, and excessive maturation of IL-1β (23, 24). This creates a state of innate immune hyper-reactivity that may lower the activation threshold of antigen-presenting cells, especially dendritic cells and macrophages in the intestinal mucosa, although direct assessment of these cellular mechanisms was not performed in the present study. In individuals who also carry HLA-DQ2 or HLA-DQ8, gluten-derived deamidated peptides are presented more efficiently to CD4+ T cells (4, 29). The result may reflect a potential interaction between innate and adaptive immune pathways: the MEFV-driven innate signals could prime the antigen-presenting machinery, while the HLA alleles may enhance the adaptive recognition of gluten, potentially leading to a stronger and more prolonged Th1/Th17 response. Direct assessment of T-cell polarization was beyond the scope of the present study (13). Our regression model identified a significant HLA-DQ2 × M694V interaction term (β = 0.38, p < 0.001), though this finding warrants cautious interpretation given that the dual genotype was present exclusively in the FMF + CD group.

Another factor that may contribute to the amplified inflammatory state is the gut microbiota. Both diseases are known to disturb intestinal microbial balance (30). In Celiac Disease, gluten exposure and genetic predisposition have been associated with dysbiosis, reduced microbial diversity, and increased intestinal permeability. This may allow bacterial products such as lipopolysaccharide to translocate into the circulation and further stimulate innate immune cells through Toll-like receptor signaling. In FMF, MEFV mutations may alter microbiota composition by dysregulating inflammasome activity, potentially favouring the growth of pro-inflammatory bacterial taxa. We speculate that when FMF and CD coexist, these microbiota-related changes may contribute together: the leaky mucosa of CD could provide a continuous source of microbial danger signals, while the hyperactive pyrin inflammasome in FMF may magnify the downstream cytokine cascade (8). The combined effect might represent a state of sustained low-grade endotoxemia driving systemic IL-6 and TNF-α production, though intestinal permeability and endotoxin levels were not directly measured in this study.

Among the cytokines we measured, IL-6 consistently stood out as the central mediator. Its strong independent association with CRP highlights its critical role in linking local mucosal events to systemic acute-phase responses (7). At the same time, the observed decrease in IL-10 levels may suggest impaired regulatory T-cell function, possibly resulting from chronic exposure to elevated IL-6 and TNF-α, though this hypothesis was not directly tested in the present study (25–27). This loss of negative feedback may contribute to the persistent and poorly controlled inflammation observed in the coexistence group. A genetic layer may also operate: IL-10 promoter variants have been linked to increased susceptibility and disease severity in both celiac disease and autoinflammatory conditions (31).

One final interpretive note concerns IL-1β. FMF is a textbook IL-1β-mediated disease, yet serum IL-1β is a notoriously unreliable proxy for inflammasome activity—it is short-lived, acts predominantly at the tissue level, and is readily suppressed by colchicine or clinical remission (7, 16, 32). Modest circulating IL-1β in our cohort should not be read as evidence of weak inflammasome engagement; rather, IL-1β serves more plausibly as an upstream initiator, with the more durable downstream products—IL-6 and TNF-α—transmitting its signal forward.

Taken together, our findings describe a distinctive immunological state in which autoinflammatory mechanisms driven by MEFV, autoimmune pathways linked to HLA-DQ2/DQ8, and microbiota-related signals may converge and potentially interact with one another (8, 13, 28). These findings may help explain the more pronounced inflammatory burden observed in patients with both FMF and CD and underscore the potential value of more personalized monitoring and treatment strategies in this overlap syndrome (11, 32).

## 5 Study limitations

We acknowledge several limitations. First, the absence of a healthy control group means we cannot directly compare our cytokine values with those of the general population. Our main focus was on clinically relevant differences between the coexistence state and single-disease states, which we consider more immediately applicable to patient care. Second, the cross-sectional design prevents any conclusion about causality or the temporal sequence of events. Longitudinal studies will be necessary to clarify these relationships. Third, assessment of gluten-free diet adherence (30) and colchicine compliance (16) relied on self-reporting. Although we used strict inclusion criteria, occasional non-adherence cannot be entirely excluded. Finally, even six months of strict gluten-free diet does not guarantee complete mucosal healing, so some degree of subclinical enteropathy may still have contributed to the observed elevations in pro-inflammatory cytokines. Additionally, disease activity indices, anti-tissue transglutaminase titres, Marsh classification scores, and longitudinal inflammatory biomarkers were not systematically available for all participants, which may have resulted in residual confounding and limits the characterisation of subclinical disease activity across groups. Disease duration, anti-tissue transglutaminase titres, and Marsh classification scores were not available for inclusion in the analysis. MEFV variant screening was not performed in the CD-only group, and HLA genotyping was recorded as a single categorical variable, which precluded analysis of HLA-DQ2/DQ8 double-positive carriers. Furthermore, detailed assay performance metrics including kit-specific detection limits, intra- and inter-assay coefficients of variation, and complete PCA outputs were not available for reporting. These gaps should be addressed in future prospective studies (4, 15).

Despite these limitations, we believe the study provides meaningful new insight into the complex interplay between FMF and CD.

## Data Availability

The data supporting the findings of this study are available from the corresponding author upon reasonable request.
